# Refugees at Work: The Preventative Role of Psychosocial Safety Climate against Workplace Harassment, Discrimination and Psychological Distress

**DOI:** 10.3390/ijerph182010696

**Published:** 2021-10-12

**Authors:** Ali Afsharian, Maureen Dollard, Emily Miller, Teresa Puvimanasinghe, Adrian Esterman, Helena De Anstiss, Tahereh Ziaian

**Affiliations:** 1 PSC Global Observatory, Centre for Workplace Excellence, Justice and Society, University of South Australia, Adelaide, SA 5000, Australia; Maureen.Dollard@unisa.edu.au; 2 Centre for Workplace Excellence, Justice and Society, University of South Australia, Adelaide, SA 5000, Australia; Emily.Miller@unisa.edu.au (E.M.); Teresa.Puvimanasinghe@unisa.edu.au (T.P.); Helena.deAnstiss@unisa.edu.au (H.D.A.); Tahereh.Ziaian@unisa.edu.au (T.Z.); 3 Clinical and Health Sciences, University of South Australia, Adelaide, SA 5000, Australia; Adrian.Esterman@unisa.edu.au

**Keywords:** psychosocial safety climate, psychological demands, harassment, psychological distress

## Abstract

It is widely recognised that employment is vital in assisting young refugees’ integration into a new society. Drawing on psychosocial safety climate (PSC) theory, this research investigated the effect of organisational climate on young refugee workers’ mental health (psychological distress) through stressful social relational aspects of work (e.g., harassment, discrimination). Drawing on data from 635 young refugees aged between 15 and 26 in South Australia, 116 refugees with paid work were compared with 519 refugee students without work, and a sample of young workers from Australian Workplace Barometer (AWB) data (*n* = 290). The results indicated that refugees with paid work had significantly lower psychological distress compared with refugees with no paid work, but more distress than other young Australian workers. With respect to workplace harassment and abuse, young refugee workers reported significantly more harassment due to their ongoing interaction and engagement with mainstream Australian workers compared with unemployed refugees. Harassment played a vital role in affecting psychological health in refugees (particularly) and other young workers. While refugee youth experienced harassment at work, overall, their experiences suggest that their younger age upon arrival enabled them to seek and find positive employment outcomes. Although PSC did not differ significantly between the employed groups, we found that it likely negatively influenced psychological distress through the mediating effects of harassment and abuse. Hence, fostering pathways to successful employment and creating safe work based on high PSC and less harassment are strongly recommended to improve refugees’ mental health and adaptation.

## 1. Introduction

By the end of 2020, an estimated 82.4 million people were forcibly displaced around the world of which about 26.4 million were refugees [1]. About half of the refugee population worldwide were under the age of 18. In Australia, the population of young (aged between 15–27) refugees and migrants have greatly increased [2,3] to over 7.6 million migrants [4]. Young refugees experience many difficulties in their migration journey, including acculturation issues and difficulty negotiating a new culture, language, and systems [5]. Some also experience identity confusion, increased parental conflict, family breakdown, and educational barriers. Others may suffer from a wide range of psychological health issues [6,7] as a result of experiencing discrimination and harassment in the settlement society [8].

Employment is commonly identified as a vital indicator of refugee integration into society [9]. Yet, one of the most common stressors for refugees and migrants is unemployment [10]. Psychological health and wellbeing deteriorate with exclusion from the workforce, with unemployed refugees reporting physical and psychological health complaints [11], lower physical health and obesity [12], depressive symptoms [13,14,15,16], lower life satisfaction [16,17], and psychological distress [17] due to financial hardship [18]. Kury and colleagues’ (2018) investigation of psychological health found that refugees (especially men) desired to work but felt overwhelmed by the culture and language barriers they encountered [7]. While both unemployment and underemployment are associated with mental health outcomes, people with mental disorders also indicate more severe symptoms if they are unemployed [19,20,21]. Clearly, refugees’ unemployment is a dilemma for both themselves and their host societies [22].

While the psychological benefits of employment are widely acknowledged, not all employment has positive benefits. There is a growing amount of literature investigating the impact of various employment-related factors such as living in rural or regional locations [23], gender [24], or country of origin [25] for refugee background workers. There is also a growing amount of literature that addresses employment aspirations [26] and barriers to and through education to employment [27] for young refugees. However, little is known about the experiences at work for young people with a refugee background. There is also increasing recognition of the importance of workplace issues (i.e., psychological disorders, verbal or physical abuse) among refugees [28,29]. Yet, little attention has been devoted to researching the factors that contribute to the employment and psychosocial risk factors at work and which may impact refugee psychological health and wellbeing. Young refugees suffer significant difficulties in obtaining employment [30] and they also encounter problems feeling a sense of belonging in the workplace, such as discrimination and harassment [31,32,33]. In this study, we explore the role of the organisational climate in the prevention and early intervention of likely stressful factors for young refugees (harassment and discrimination) that could be related to mental health and psychological distress.

This paper is an exploratory study: (1) comparing the psychological distress of young refugees (engaged in paid work or unemployed) and non-refugee young workers (data derived from pathways to active citizenship project), (2) examining whether work is a more negative experience for refugees vs. other young workers (data derived from Australian Workplace Barometer project), and (3) examining how the organisation’s Psychosocial Safety Climate (PSC) functions in relation to psychological demands and harassment that refugees may experience at work to predict psychological distress across industries in South Australia. We focus on young refugees because there is evidence that they face a distinctive set of challenges [34] that place them at increased risk of unemployment that are outside the experience of youth in the general population [35].

## 2. Theoretical Framework: Psychosocial Safety Climate

In this paper we frame the experience of psychological distress at work in terms of the Psychosocial Safety Climate (PSC) framework. PSC is a facet of organisational climate and concerns the environment for worker psychological health. It is characterised by management commitment to stress prevention, management priority for worker psychological health over productivity concerns, organisational communication, and systems to inform about factors that affect psychological health and participation of all stakeholders in resolving issues that affect psychological health. PSC largely emanates from senior management values and actions that affect working conditions and social relations and is likely important for the psychological health and wellbeing of young refugee workers at work.

There is robust evidence that shows PSC can predict employees’ health along with motivational outcomes such as work engagement. PSC is an important indicator of emotional exhaustion and psychological distress [36,37,38], emotional exhaustion and cynicism [39], depression [40,41], work engagement [39,42], sickness absence in workers [36,43], employee presenteeism [43], and compensation claims for physical injuries at work [40,44,45]. In sum, PSC is a leading indicator of work conditions and social–relational aspects (harassment and discrimination), health and motivational outcomes, and productivity.

### 2.1. Workplace Harassment and Abuse

The migration journey is not easy nor straightforward [46] and can involve continued periods of hardship and suffering [47], including mental health issues and challenges [48,49,50,51,52]. Earlier studies investigating depression, and emotional and behavioural problems among refugee children and adolescents living in South Australia found that reported symptoms rates were significantly lower than those reported for young refugees in other resettlement countries [53,54,55]. Nonetheless, the challenges of pre- and post-settlement can negatively influence their overall wellbeing compared with Australian-born equals [24,56,57]. 

Migrants usually experience stressful situations such as being discriminated against because of their different ethnic origin, which makes them feel unfairly treated [58]. Harassment is defined as intimidating, offending, or humiliating behaviours that target personal attributes such as race, culture, nationality, religion, age, disability, gender, or sexuality [59]. Examples of harassment and discrimination investigated in this research are negative comments made about a person’s ethnic or racial background, unwanted sexual advances, being sworn at or yelled at, being humiliated in front of others, being physically assaulted/threatened and sexual humour and unfair treatment due to gender. 

We expect that:

**Hypothesis** **1.** *Young refugee workers will report significantly more harassment and abuse (i.e., gender, sexual, racial or ethnic harassment, and abuse, being humiliated in front of others or being sworn at, and/or being physically attacked/threatened) in the workplace compared with non-refugee workers (young Australian workers from the Australian Workplace Barometer (AWB) dataset)*.

### 2.2. Health Effects of Employment 

Since employment can bring benefits such as income, financial security, meaning, and structure to life [60], having a job is expected to be better for psychological health than not having a job.

We expect that:

**Hypothesis** **2.** *Young refugee workers will report significantly lower levels of psychological distress compared with young refugees who are not working*.

Experiencing stressful environments can severely affect psychological health since working contexts are tightly linked to the risk of mental health issues and psychological distress [61].

Since harassment is a known psychosocial risk for psychological distress and young refugee workers are likely to report more harassment than non-refugee workers, we expect that:

**Hypothesis** **3.** *Young refugee workers will report significantly more psychological distress compared with non-refugee workers (young Australian workers from the Australian Workplace Barometer (AWB) dataset)*.

### 2.3. Psychosocial Safety Climate, Harassment and Psychological Distress

PSC reflects the extent that workers’ psychological health and safety is valued in the workplace. In working environments with high PSC, a pro-social focus has been embodied, and we expect a better quality of work, enhanced meaningfulness, more innovation and creativity, and less productivity loss due to sickness absence and presenteeism. PSC is considered the ‘cause of the causes’ of work stressors, and at low levels, results in poor work quality (high demands, low resources) and greater social–relational risks (such as bullying and harassment).

Managers in high PSC contexts consider the mental health of employees to be as important as productivity and as such, support and protect psychological health. Therefore, in a high PSC working environment, employees find job demands to be more manageable, are likely to have high control and more opportunities to learn and fulfill their psychological needs [62]. In low PSC contexts, different elements such as low quality of work, high demands, and low control, or tedious work, are likely to threaten and thwart the psychological fulfilment of work and result in mental health issues [62]. PSC is also important for providing signals to workers on social–relational characteristics of work like how workers should relate to each other, and the types of behaviours rewarded or sanctioned such as whether discrimination, workplace bullying, and harassment will be tolerated [62]. Many studies have found that PSC negatively predicts demands [39,62,63], and bullying and harassment at work [42], and positively predicts resources [36,39]. PSC is negatively linked to psychological distress in a number of ways. First, it is an upstream factor that predicts a range of psychosocial risk factors (i.e., psychological demands and harassment). It is through these risk factors that PSC could relate to psychological distress. According to the conservation of resources theory [64], workers are motivated to build, maintain, and conserve the resources. Any threats to resources may cause stress to occur. The lowest loss of the psychological resources at work will be ensured in high PSC working environments. Consequently, in high PSC settings workers can advance their safety behaviours since their psychological health concerns are decreased [65]. Thus, workers can preserve the resources to invest in cultivating the safety behaviours at work without being concerned about their psychological health, and the imperative psychological resources will be secured.

Second, PSC relates to psychological health through workplace communication and participation processes. Based on PSC values, managers ensure essential policies are in place, such as prioritising health and safety over productivity, strengthening their organisational commitments toward mental health and safety at work and applying effective communication and participation systems to address employee concerns. In this way, any potential threat to psychological health could be removed or ameliorated, thus linking PSC to psychological health status. Thus, we propose that:

**Hypothesis** **4.** *Young refugee workers will report significantly lower levels of PSC compared with other employed samples in Australia*.

Finally, on an exploratory basis, we investigated whether PSC as a distal variable is negatively associated with psychological distress via its relationship with workplace harassment and abuse.

## 3. Materials and Methods

### Participants and Procedure

Full sample: Pathways to active citizenship data include both qualitative (112 individual interviews with young refugees, parents, and teachers) and quantitative data collected from young refugee participants (*n* = 635; male 40.3%, female 59.7%) aged between 15–27 (Mean = 20; Ziaian et al., 2018). Participants were selected based on the following criteria: (1) 1 to 15 years of Australian residence, (2) arrived from one of three key migration regions: Africa, South Asia, or the Middle East, and (3) being currently enrolled in formal study. Reading ability was not assessed as it was expected that all the participants would be students and have enough English language proficiency to complete the survey. However, Bilingual Youth Workers (BYWs) who spoke the same language and came from the same or similar ethnic background as the participants were recruited to clarify any potential confusion related to language or cultural barriers among participants and guide participants through survey completion. Participants also had the option of completing the survey in English or their native language with support from an official interpreter andusing the bilingual approach of having BYWs guide participants by offering language and cultural assistance when requested, enhanced communication and ensured survey completion. Participants who completed the survey were compensated AUD 15.00 for their participation.

Young refugee worker sample. Most participants (81.7%) were engaged in formal education (either high school, Technical and Further Education—TAFE or university), and 116 (18.3%) were in paid work. Of the 116 in paid work, 54 were males, and 62 were females aged between 15–26 years, with a mean of 20.43 years (SD = 2.73). Nearly two-thirds of the participants (83.6%) were aged 18 and above.

Young Australian worker sample. To compare with a national sample of young workers in Australia, a matched age group of 290 young workers (152 males and 138 females aged 18–26 years with a mean of 22.3 years old (SD = 2.65) were selected from a larger AWB dataset and included in the study.

## 4. Measures

### 4.1. PSC

PSC was measured using the PSC-4 [62], a 4-item questionnaire encompassing four sub-scales: Management commitment and support, management priority, organisational communication, and participation [66]. Example items for each subscale, respectively, are: (1) ‘Senior management shows support for stress prevention through involvement and commitment’, (2) ‘Senior management considers employee psychological health to be as important as productivity’, (3) ‘There is good communication here about psychological safety issues which affect me’, and (4) ‘In my organisation the prevention of stress involves all levels of the organisation’. Each sub-scale consists of one question with a response range scored on a five-point Likert scale, from 1 = strongly disagree to 5 = strongly agree (α = 91).

To determine the status of one’s workplace with respect to PSC, benchmarks have been determined [40]. PSC-12 scores range from 12–60, with low-risk PSC (scores ≥ 41), medium-risk PSC (scores 41< and >37), high-risk PSC (scores 37≤ and >26) and very high-risk PSC (≤26) for predicting job strain and depression. Likewise, a PSC raw score of 26 and less signals the urgent need for mental health intervention and prevention of employees’ depression [67].

### 4.2. Workplace Harassment and Abuse Scale

Workplace harassment and abuse was measured using the Richman et al. (1996) eight-item scale, including gender, sexual, racial or ethnic harassment and abuse, being humiliated in front of others or being sworn at, and/or being physically attacked/threatened [59]. Responses were scored using a 5-point Likert scale, ranging from very rarely/never = 1 to very often/always = 5 (α = 0.98).

### 4.3. Psychological Distress

Psychological distress was measured using the Kessler 10 (K10) [68]. All ten items were included to explore anxiety and depression levels and symptoms that the employees had experienced during the last four weeks. Responses were scored using a 5-point Likert scale, ranging from none of the time = 1, to all of the time = 5 (α = 0.86).

## 5. Data Analysis

Statistical analysis was conducted using SPSS-26 [69] and AMOS [70]. Prior to this, the NMISS function was applied to return the number of missing values over the variables before coding and mean replacement. Thus, only participants who had responded to at least 70% of the questions in each scale were included in the preliminary and principal analyses. Means (M), standard deviations (SD), and correlations between variables drove the descriptive statistics. T-tests were performed to show differences between mean levels of harassment, distress, health and PSC. Since specific aspects of harassment and abuse are important to understand, we interpreted responses to that scale on an item-by-item basis. A chi-squared test was applied to assess the association of the PSC benchmarks across the youth refugee workers and other working groups. The mediating effect of job demands (psychological) and harassment in the relation between PSC and psychological distress is illustrated using R software.

## 6. Results

The intercorrelations of variables are shown for young refugee and Australian worker samples in Table 1.

Means, standard deviations, and *t*-test or one-way ANOVA results are shown for the young refugee and Australian worker samples in Table 2.

Hypothesis 1 predicted that young refugee workers would report significantly more harassment and abuse (i.e., gender, sexual, racial or ethnic harassment and abuse, being humiliated in front of others or being sworn at, and/or being physically attacked/threatened) than non-refugee young Australian workers. As shown in Table 2, there was a significant difference in levels of harassment reported by refugee vs. other young Australian workers. Refugee workers reported significantly higher levels of harassment, in particular, more sexual harassment, discomfort listening to sexual humour, more unfair treatment due to gender, more negative comments about ethnicity, more humiliation and more physical violence from organisational members and clients/patients. The only item where there was no difference between the groups was being sworn at. For example, the results indicated that refugee participants reported significantly higher levels of unwanted sexual advances (1.31 ± 63) compared with Australian workers (1.05 ± 27), *t* (404) = 5.904 (See Table 3). Therefore, Hypothesis 1 was supported.

Hypothesis 2 proposed that young refugee workers would report significantly lower levels of psychological distress compared with young refugees who are not working, and as shown in Table 2, this was supported.

The results indicated that young refugee participants with paid jobs had significantly lower psychological distress (18.43 ± 6.78) compared with young refugees without jobs (20.26 ± 8.01), *t* (565) = −2.217, Cohen’s *d* = 0.245. Comparing multiple groups, refugee workers, non-working refugee workers and young Australian workers, the one-way ANOVA results indicated that non-working refugees reported significantly higher levels of psychological distress compared with refugee workers, and Australian workers (15.95 ± 5.95), F (854) = 32.52 (See Table 2).

Hypothesis 3 proposed that youth refugee workers would report significantly more psychological distress than non-refugee workers (young Australian workers from the Australian Workplace Barometer (AWB) dataset). As shown in Table 2, this was also supported.

Hypothesis 4 proposed that young refugee workers would report significantly lower levels of PSC compared with other employed samples in Australia. Note, only 108 refugee participants completed the surveys on the PSC-4 scale. To compare the results with the PSC benchmarks [40], PSC-12 was computed by multiplying PSC-4 into 3 in advance. For the 108 young refugee employees, even though most described their working environments with high PSC (61.1%), there were 5.6% in medium-risk contexts, 25.9% in high-risk contexts, and 7.4% in very high-risk PSC contexts which indicate very low PSC level (See Table 4).

There was no significant association between the PSC risk levels and the various work samples. Specifically, there was no significant differences between the PSC risk levels for young refugees compared with: other Australian youth employees, chi-squared (3) = 4.52, *p* = 0.21; the total sample of working population in South Australia, chi-squared (3) = 1.49, *p* = 0.70; and the national sample, chi-squared (3) = 6.26, *p* = 0.10 (see Table 4). Therefore, Hypothesis 4 was not supported as there was no association between group type and PSC levels.

Finally, on an exploratory basis, we assessed the process via which PSC is negatively associated with psychological distress, exploring this relationship via workplace harassment and abuse. The multigroup modelling shown for the unconstrained model indicated a very good fit on GFI, but the fit on other indices were not as good as expected. When the models were constrained to have equal weights between the variables the model fit was not significantly improved, indicating that the size of the weights across samples was equivalent (see Table 5). We tested competing models, including a main effects model with PSC and harassment/abuse both in direct association with distress. There are six possible combinations of the three variables. In SEM a reverse model of any combination will yield exactly the same model fit. We chose two exemplars of alternative paths aside from PSC-H→PD (because this would be the same). As can be seen in Table 5, these alternative models were not a better fit than the PSC→H→PD model.

Further, since we had theory behind this formulation, we proceeded with the PSC→H→PD model.

**Figure 1:**
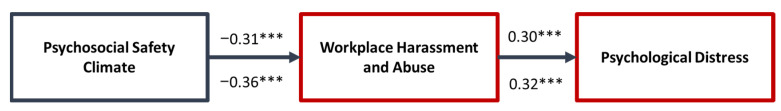
PSC effect on psychological distress via mediating role of workplace harassment and abuse. ***, *p* < 0.001.

As can be seen in Table 6, PSC negatively and significantly predicted workplace harassment and abuse (Beta = −0.31 ***) and harassment and abuse were significantly positively related to psychological distress (Beta = 0.30 ***). The indirect effect was significant, Beta = −0.09, Lower Bound = −0.17, Upper Bound = −0.05, *p* = 0.002). Likewise, for the young Australian worker, the mediation/ indirect effect was also significant (see Figure 1).

## 7. Discussion

Employment is critical to both economic and social participation and the long-term integration of young refugees. Their successful integration as fully participating and included Australian citizens is desirable for them and Australian society at large [35]. In this study, we used PSC theory to frame and understand young people’s experiences of work pressure, organisational harassment, and psychological distress whilst employed in different industries across South Australia. We sought to determine whether employment could confer positive psychological health effects on young refugee workers and consider which social relational aspects of work, specifically harassment and abuse, could negate the positive benefits of employment. We found that work did have a positive benefit for young refugee workers since their levels of psychological distress were significantly lower than an age-matched sample of non-working refugees. However, compared with non-refugee samples, the young refugee workers had significantly higher levels of distress, highlighting potential exposure to different stressors. The higher levels of psychological distress could be explained in terms of the significantly higher levels of harassment and abuse that they experienced compared with other young Australian workers. Slightly at odds with this, the corporate climate for psychological health (PSC), was perceived similarly by all groups of workers, young refugees and other Australian workers.

Our research found that PSC is negatively related to workplace harassment and abuse, and this in turn gives rise to psychological distress. In low PSC contexts characterised by a lack of concern about worker psychological health, it is likely that there is no signalling about appropriate respectful behaviours. Across the young worker groups, PSC was negatively related to the experience of harassment and abuse, indicating that this relationship is generalisable across the samples. Likewise, the mediation process was also evident across both samples, thus the process of lower PSC giving rise to harassment and abuse in turn leading to psychological distress is not specific to the refugee group. However, the refugee group did experience greater harassment and abuse and distress. While young refugees in Australia have been noted to face these barriers to employment, and although these challenges may negatively impact young people’s education, employment, and mental health, overall, their academic and psychosocial resilience and adaptability is well documented [71,72]. Young refugees work hard to achieve employment aspirations which are connected to hopes for the future, settlement experiences, and systems of support that enable these processes [26,73]. In a previous study by Ziaian, et al. (2012), only 5.6% of refugee children and adolescents met the criteria for depression [55], a figure that is only slightly higher than that reported for children and adolescents in the general Australian population [74].

Previous research has also highlighted that young refugees with higher levels of resilience experience fewer mental health problems [54]. In order to find a job, refugee youth need a certain level of resilience in addition to English language skills and an ability to navigate the Australian employment system. Those who are already employed may experience the same level of psychological distress or perceive discrimination compared with non-working refugees despite their job challenges and stressful work environment.

This may be due in part to the gratitude and optimism that working refugees often express after securing stable employment in their country of resettlement [35,75,76]. This positivity and optimism may decrease job stress even though the work environment may be stressful. Another possible explanation is that working refugees may have underreported psychological distress due to their employment vulnerability.

### 7.1. Practical Implications

Positive employment experiences can enhance the active citizenship of young people from refugee backgrounds by fostering a sense of belonging and enhancing integration into their new country, improving their economic status and reducing psychological distress [77]. To this end, a supportive working climate like PSC can signify the importance of support for health and wellbeing at work, communication regarding workplace health and wellbeing issues and prevention of stress by improving the quality of the work environment. Therefore, in order to significantly improve the work health and safety conditions, psychological health, and work engagement, and yield profits in terms of work productivity, it is essential to focus on enhancing PSC in organisations.

### 7.2. Limitations and Suggestions for Future Research

Using a convenience sampling method to recruit participants is a potential limitation of this study as it may limit the generalisability of the results. We used a convenience sampling method due to the unavailability of a representative sampling frame. Despite the fact that anonymity and confidentiality were well explained, and a large number of young refugees sufficiently trusted the process to volunteer their participation, nonetheless, some potential participants may have declined to participate for fear that it may lead to questions about their immigration status or for other reasons. Previous research has found higher rates of distrust and suspicion towards research among refugees [56,57].

The data obtained may be subject to social desirability bias as some participants requested and were assisted by BYWs to complete the survey. The presence of a BYW may have influenced participants’ survey responses. A final limitation of the current study is the cross-sectional nature of the data. Causal mediation relationships between PSC, harassment and distress cannot be ‘causally’ verified with cross-sectional data. Future longitudinal research can investigate whether PSC influences changes in harassment and abuse, whether these changes influence changes in psychological distress over time, assess how long associations between the measures take to emerge, and assess potential reverse impacts of psychological distress on PSC perceptions, and whether being in a state of psychological distress makes one vulnerable to harassment and abuse.

With respect to future research, conducting a multi-level study is recommended with a larger sample to investigate PSC in different industries since PSC is a concept that emerges at higher organisational levels (i.e., teams, workgroups, and organisations).

Future studies could also examine whether there are any differences in the relationships between PSC, harassment and distress among young people from refugee backgrounds permanently resettled in Australia, young refugees on temporary visas, and asylum seekers, and the extent to which PSC moderates the relationships between job demands and psychological distress. Another contribution would be to investigate trauma prior to migration to determine the chronic effects of this on subsequent adaptation.

As the relationships between PSC, harassment, abuse, and distress may vary across refugee and migrant communities, and other psychosocial risk factors other than harassment and abuse may be experienced, a qualitative study focusing on perceptions and understanding of workplace policies and practices like PSC can identify specific workplace needs of these communities.

## 8. Conclusions

Typically, young refugees experience various psychological, emotional, and social, economic challenges as they seek to rebuild their lives in Australia. As employment can be linked to psychological distress, focusing on psychosocial risk factors through the lens of PSC can reduce psychological demands and workplace harassment. The results of this study support previous findings [62] that PSC affects job demands (psychological) and that harassment and abuse can predict workers’ psychological distress. The results highlight the role that work environments with high PSC play in assisting young refugees to maintain their employment and successfully integrate in Australia.

## Figures and Tables

**Table 1:** Intercorrelations among variables.

	1	2	3	4	5
1. Gender	-	−0.09	−0.04	0.01	0.09
2. Age	−0.14	-	−0.08	0.02	−0.03
3. PSC	0.02	−0.09	-	−0.35 **	−0.35 **
4. Workplace Harassment	−0.09	−0.16	−0.33 **	-	0.32 **
5. Psychological Distress	0.14	−0.16	−0.10	0.35 **	-

Note: Correlations above the diagonal line belong to the young Australian worker sample, and the below the diagonal line belong to the young refugee worker sample. M = mean, SD = standard deviation, n = number of participants who completed the scale, ** Correlation is significant at the 0.01 level (2-tailed).

**Table 2:** *T*-tests and one-way ANOVA for comparing refugee workers, refugee non-workers and non-refugee workers (AWB) on levels of psychological distress, harassment, and Psychological Safety Climate (PSC).

	Refugee				Non-Refugee		t/F ^†^ (*df*)	*p*	d
	Worker		Non-Workers		Workers				
	M	SD	M	SD	M	SD			
Harassment	12.15	5.09	N/A	N/A	9.78	2.87	5.92 ** (404)	<0.001	0.574
Psychological distress	18.43	6.78	20.26	8.01	15.95	5.59	−2.17 * (565) 32.52 **^,†^ (854)	0.03 <0.001 ^†^	0.245 N/A ^†^
PSC	14.49	3.83	N/A	N/A	13.88	3.42	1.53 (389)	0.13	0.168

Note. d = Cohen’s d, M = mean, SD = standard deviation, *df* = degree of freedom, **^†^** = one-way ANOVA, ** = *p* < 0.01 level, * = *p* < 0.05 level.

**Table 3:** *T*-tests for comparing refugee workers vs. non-refugee workers (AWB) on levels of workplace harassment and abuse.

	Young Workers	N	Mean	SD	t	*p*
Unwanted sexual advances	Refugee	116	1.31	0.63	5.90	<0.001
	Australian	290	1.05	0.27	4.35	<0.001
Discomfort listening to sexual humour	Refugee	116	1.48	0.79	2.81	0.01
	Australian	290	1.26	0.69	2.65	0.01
Experience unfair treatment due to gender	Refugee	116	1.52	0.87	4.24	<0.001
	Australian	290	1.19	0.63	3.71	<0.001
Negative comments about my ethnicity	Refugee	116	1.72	0.93	8.99	<0.001
	Australian	290	1.11	0.43	6.75	<0.001
Been sworn at	Refugee	116	1.76	0.93	1.38	0.17
	Australian	290	1.62	0.98	1.41	0.16
Been humiliated	Refugee	116	1.54	0.80	3.42	<0.001
	Australian	290	1.27	0.67	3.17	<0.001
Been physically assaulted/threatened by a member of organisation	Refugee	116	1.42	0.86	6.38	<0.001
	Australian	290	1.05	0.32	4.57	<0.001
Been physically assaulted/threatened by client or patient	Refugee	116	1.40	0.82	2.11	0.04
	Australian	290	1.24	0.63	1.89	0.06
Harassment (total)	Refugee	116	12.15	5.09	5.93	<0.001
	Australian	290	9.78	2.87	4.73	<0.001

Note: degree of freedom (*df*) = 404.

**Table 4:** PSC-12 Benchmark standards, classification for young refugees in comparison with AWB data (youth national, SA employees, and the total national sample).

PSC Standards	Range 12–60	Young Refugee Employees (2018) *n* = 108 Number (%)	Young Employee National Sample (AWB 2014–15), *n* = 290 Number (%)	SA Employees (AWB 2014–15), *n* = 325 Number (%)	National Employee Sample (AWB 2014–15), *n* = 3736 Number (%)
Low-risk PSC (High PSC)	≥41	66 (61.1)	172 (66.4)	208 (63.9)	2092 (56.0)
Medium-risk PSC	41< and >37	6 (5.6)	18 (6.9)	25 (7.7)	362 (9.7)
High-risk PSC	37≤ and >26	28 (25.9)	47 (18.1)	69 (21.4)	851 (22.8)
Very high-risk PSC (Very low PSC)	≤26	8 (7.4)	22 (8.5)	23 (7.0)	430 (11.5)

Note. AWB, Australian Workplace Barometer.

**Table 5:** Fit measures.

Model	GFI	CFI	IFI	NFI	RMSEA	Χ^2^	df	Χ^2^ Change	*p*
Unconstrained	0.97	0.82	0.83	0.81	0.155	21.49	2		
Constrained weights	0.96	0.83	0.82	0.79	0.111	24.06	4	2.57	0.28
Structural covariances	0.95	0.82	0.82	0.79	0.116	24.06	5	10.69	0.013
Structural residuals	0.85	0.14	0.13	0.12	0.182	20,118	7	79.69	<0.001
**Alternative**									
Main effects	0.93	0.54	0.55	0.54	0.25	52.63	2		
H→PSC→PD	0.96	0.80	0.81	0.80	0.16	23.47	2		
H→PD→PSC	0.95	0.79	0.71	0.70	0.20	34.96	2		

**Table 6:** Mediation results.

			Refugee Worker				Young Australian Worker			
			***Beta***	***B***	**SE**	***t***	***Beta***	***B***	**SE**	***t***
PSC	→	Harass.	−0.31	−0.14	0.04	−3.52 ***	−0.36	−0.12	0.02	−6.63 ***
Harass.	→	Psych. Distress	0.30	0.38	0.14	3.35 ***	0.32	0.62	0.11	5.67 ***
			***Beta***	**LB, UB**	**SE**	***p***	***Beta***	**LB, UB**	**SE**	***p***
Indirect effect PSC→ Psych. Distress			−0.09	−0.17, −0.05	0.03	0.002	−0.12	−0.16, −0.06	0.04	0.015

Note: ***, *p* < 0.001, LB, lower bound, UB, upper bound.

## Data Availability

The AWB data is available from the Australian Data Archives at the Australian National University. The Pathways to Active Citizenship data are not in the public domain due to the sensitivity of the research area and the vulnerability of the population concerned. However, all data have been stored in the University of South Australia’s Data Storage Centre and will be available for 5 years, to research team members, if required.
